# Genomic expression dominance in allopolyploids

**DOI:** 10.1186/1741-7007-7-18

**Published:** 2009-05-01

**Authors:** Ryan A Rapp, Joshua A Udall, Jonathan F Wendel

**Affiliations:** 1 Department of Ecology, Evolution and Organismal Biology, Bessey Hall, Iowa State University, Ames, Iowa 50010, USA; 2 Department of Plant and Wildlife Sciences, WIBD, Brigham Young University, Provo, Utah 84602, USA

## Abstract

**Background:**

Allopolyploid speciation requires rapid evolutionary reconciliation of two diverged genomes and gene regulatory networks. Here we describe global patterns of gene expression accompanying genomic merger and doubling in inter-specific crosses in the cotton genus (*Gossypium *L.).

**Results:**

Employing a micro-array platform designed against 40,430 unigenes, we assayed gene expression in two sets of parental diploids and their colchicine-doubled allopolyploid derivatives. Up to half of all genes were differentially expressed among diploids, a striking level of expression evolution among congeners. In the allopolyploids, most genes were expressed at mid-parent levels, but this was achieved via a phenomenon of genome-wide expression dominance, whereby gene expression was either up- or down-regulated to the level of one of the two parents, independent of the magnitude of gene expression. This massive expression dominance was approximately equal with respect to direction (up- or down-regulation), and the same diploid parent could be either the dominant or the recessive genome depending on the specific genomic combination. Transgressive up- and down-regulation were also common in the allopolyploids, both for genes equivalently or differentially expressed between the parents.

**Conclusion:**

Our data provide novel insights into the architecture of gene expression in the allopolyploid nucleus, raise questions regarding the responsible underlying mechanisms of genome dominance, and provide clues into the enigma of the evolutionary prevalence of allopolyploids.

## Background

Polyploidy is prevalent in nature and is particularly common in the angiosperms, where it is both an ancient and active evolutionary process [1-3]. In the past decade expressed sequence tag (EST) and genome sequencing projects revealed numerous rounds of ancient polyploidy scattered throughout the angiosperms [4-6], confirming and expanding upon more than a century of comparative cytogenetic work [7,8], which demonstrated that polyploidy is common and ongoing in hundreds of genera. In plants, polyploidy often is associated with novel and presumably advantageous ecological attributes, such as range expansion [9], novel secondary chemistry and morphology [10], and increased pathogen resistance [11], although the underlying genetic basis for these novel adaptations remains obscure. The reunion of two diverged genomes in a common nucleus during allopolyploid speciation entails a suite of genomic accommodations [12-14], including non-additivity of gene expression [15,16], and expression partitioning among tissues and organs [17-20]. Of particular interest are the mechanisms by which doubled regulatory networks interact to generate a viable genetic system capable of regulating growth, development and responses to the environment.

To better understand the earliest stages of allopolyploid evolution, we monitored gene expression in two sets of diploid parents and their colchicine-doubled allopolyploid derivatives from the genus *Gossypium *(L.), which has become a useful model for polyploid evolution [19-21]. Our goal was to determine the effects of genomic merger and doubling on global gene expression architecture. To our surprise, we discovered in both crosses a striking pattern of 'expression dominance', where gene expression for thousands of genes closely mirrored that of only one of the two parents, both for up-regulated and down-regulated genes. In addition, we also detected a diverse spectrum of transgressive gene expression types and levels. Collectively, these results provide a novel perspective on allopolyploid gene regulation and hint at the underlying genetic basis of allopolyploid adaptation.

## Results and discussion

**Figure 1:**
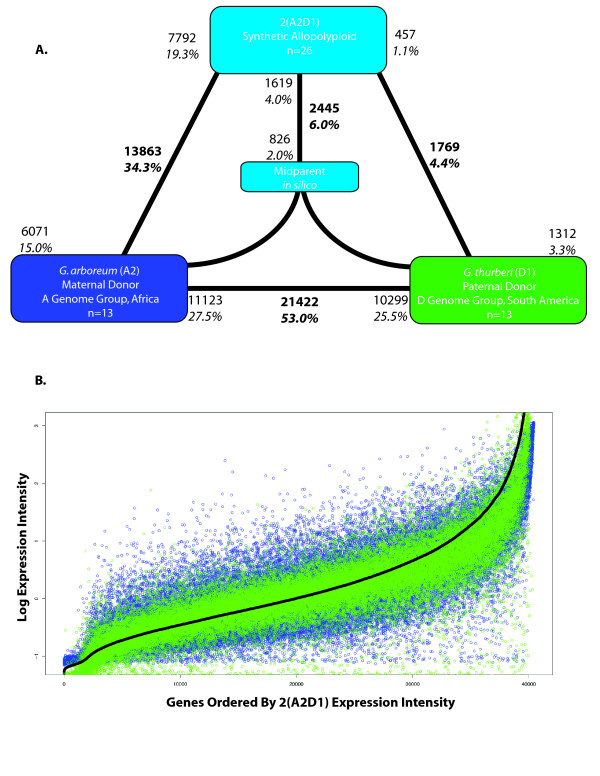
**Differential expression and genome-wide expression dominance in *Gossypium *allopolyploids**. **A**. Synthetic allopolyploid 2(A2D1) generated from the diploid parents *G. arboreum *and *G. thurberi*. *G. arboreum *was the maternal parent in each cross. Bold text indicates the total number and fraction of genes diagnosed as differentially expressed in each contrast. Also shown for each contrast is the partitioning of the total number of differentially expressed genes into the direction of up-regulation; these numbers are indicated by the non-bold text. For example, in panel **A**, 13,863 genes are indicated as being differentially expressed between *G. arboreum *and the synthetic allopolyploid. Of these, 7792 were up-regulated in the synthetic allopolyploid, and 6071 were up-regulated in *G. arboreum*. Differential expression between each allopolyploid and its *in silico *mid-parent is shown in the middle of each triangle. Around 53.0% of the 40,430 unigenes were differentially expressed between diploids, with a range of 4.4% to 34.3% between diploids and their allopolyploid derivatives. Expression dominance is illustrated by the eight-fold asymmetries in differential expression between the allopolyploids and their diploid antecedents. **B**. Genes were ordered by their normalized, standardized expression intensity in the allopolyploid (black line). The expression intensity of the parents was superimposed (maternal = blue; paternal = green) to illustrate the expression level similarity between the dominant genome and the allopolyploid. Thus, variance around the green dots (the dominant genome) is lower than around the blue dots. Thus, expression dominance is bidirectional with respect to the status of the *G. arboreum *expression phenotypes in the two allopolyploids.

**Figure 2:**
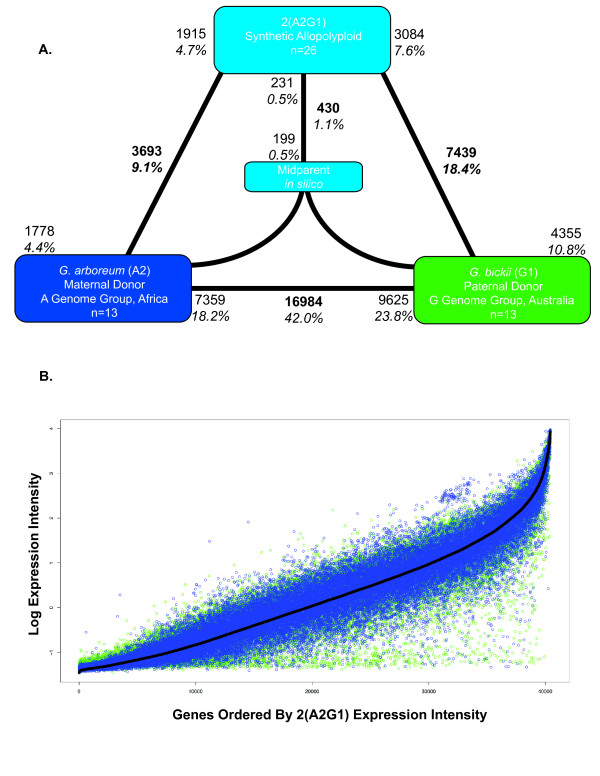
**Differential expression and genome-wide expression dominance in *Gossypium *allopolyploids**. **A**. Synthetic allopolyploid 2(A2G1) generated from the diploid parents *G. arboreum *and *G. bickii*. *G. arboreum *was the maternal parent in each cross. Bold text indicates the total number and fraction of genes diagnosed as differentially expressed in each contrast. Also shown for each contrast is the partitioning of the total number of differentially expressed genes into the direction of up-regulation; these numbers are indicated by the non-bold text. Differential expression between each allopolyploid and its *in silico *mid-parent is shown in the middle of each triangle. Around 42.0% of the 40,430 unigenes were differentially expressed between diploids, with a range of 4.4% to 34.3% between diploids and their allopolyploid derivatives. Expression dominance is illustrated by the two-fold asymmetries in differential expression between the allopolyploids and their diploid antecedents. **B**. Genes were ordered by their normalized, standardized expression intensity in the allopolyploid (black line). The expression intensity of the parents was superimposed (maternal = blue; paternal = green) to illustrate the expression level similarity between the dominant genome and the allopolyploid. Thus, variance around the blue dots (the dominant genome) is lower than around the green dots. Thus, expression dominance is bidirectional with respect to the status of the *G. arboreum *expression phenotypes in the two allopolyploids.

To monitor gene expression in nascent allopolyploids we grew two sets of diploid parents and their colchicine-doubled allopolyploid derivatives in a randomized complete block design in three separate growth chambers. We hybridized labeled leaf cDNAs to custom micro-arrays, assaying 40,430 genes for their relative expression levels, and determined for each gene the level of expression variation between the parents, between each parent and the allopolyploid, and between the *in silico *mid-parent expression value and the allopolyploid. Here, we take mid-parent to mean the average of the empirically determined parental values, across all replicates (see the methods section). The general levels of differential expression in these experiments are illustrated in Figures 1 and 2.

We first evaluated differential expression between the two diploid parents involved in each cross (*G. arboreum *(A_2_) and *G. bickii *(G_1_); *G. arboreum *(A_2_) and *G. thurberi *(D_1_)), postulating that the degree of parental divergence would be correlated with the amount of non-additivity in their respective synthetic allopolyploid (2(A_2_D_1_) and 2(A_2_G_1_)). As all plants were grown under common controlled conditions, we expected only modest expression differentiation among diploids, but high levels of expression divergence were observed; 42.0% and 53.0% of the 40,300 unigenes were differentially expressed between *G. arboreum *(A-genome group) and *G. bickii *(G-genome group), and *G. arboreum *and *G. thurberi *(D-genome group), respectively (Figures 1 and 2, panels A). The larger difference in the latter comparison is consistent with data showing that the A and G genomes are more similar in size and are phylogenetically closer to each other than either is to the D genome [21]. All three species are shrubs native to arid regions but are from three different continents (*G. arboreum *from Africa, *G. bickii *from Australia, and *G. thurberi *from North America), having diverged from a common ancestor approximately 5 to 10 million years ago [21]. Their extraordinary gene expression divergence was unexpected given an average coding sequence divergence of about 3% [22], and represents the greatest divergence reported to date among congeneric plant species [16,23]. Among the differentially expressed genes, equivalent proportions are up-regulated in each parent (18.2% (*G. arboreum*, A_2_) versus 23.8% (*G. bickii*, G_1_) and 27.5% (*G. arboreum*, A_2_) versus 25.5% (*G. thurberi*, D_1_); *P *> 0.05 in *χ*-square tests).

To assess the impact of combining two diverged regulatory networks on gene expression in allopolyploids, we contrasted each parent with the allopolyploid, and the allopolyploid with an *in silico *mid-parent value, generated using an average of the parental values and a composite variance. A high fraction of genes were differentially expressed between the allopolyploids and the parental diploids (27.5% and 38.7%, in 2(A_2_G_1_) and 2(A_2_D_1_), respectively). Also, and perhaps as expected, most genes in the allotetraploid were expressed at values equivalent to the mid-parent, namely, 99.0% and 93.9% in 2(A_2_G_1_) and 2(A_2_D_1_), respectively.

**Figure 3:**
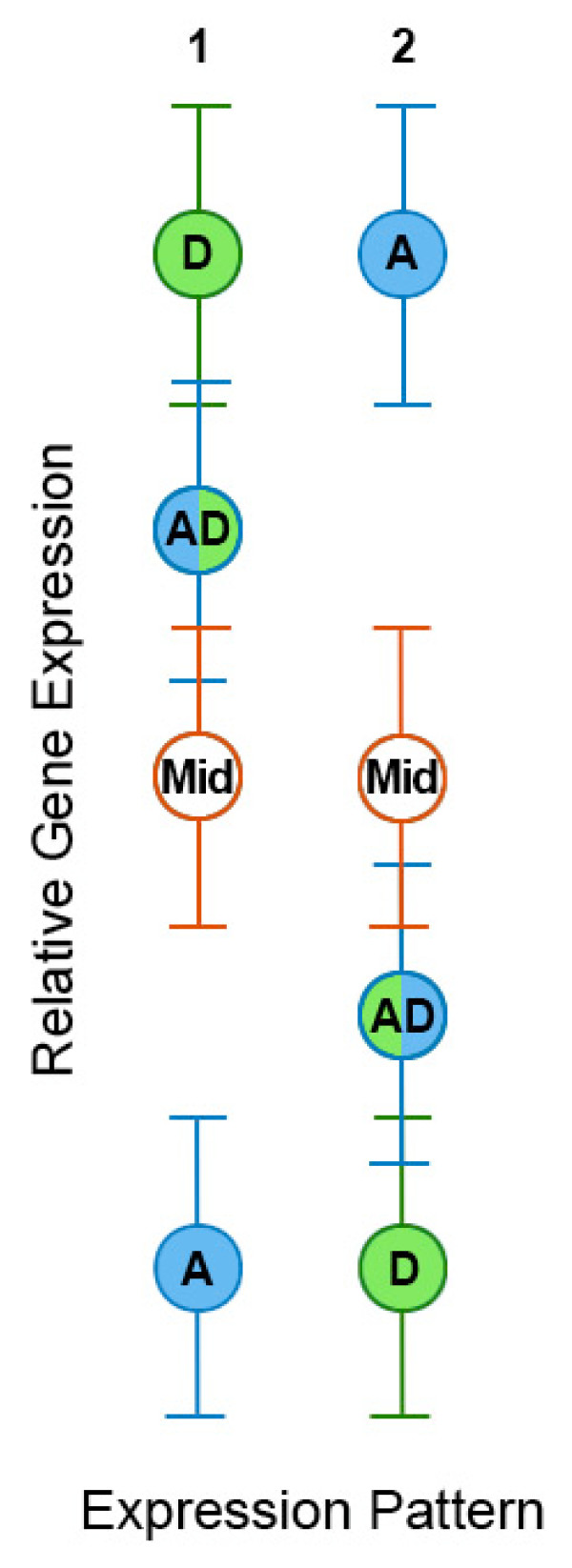
**Statistical interpretations of differential expression between parental diploids, their allopolyploid derivative, and an *in silico *mid-parent**. The vertical axis represents relative expression level. Each taxon is labeled and color-coded with its corresponding error bar. Lanes 1 and 2 represent two separate genes with expression patterns like those in Figure 4 panels XI and II, respectively. For the first gene (lane 1), expression is higher in the D-genome than in the A-genome diploid parent. Expression in the D genome is statistically equivalent to that of the allopolyploid but not to the mid-parent (indicated by non-overlapping error bars), while expression in the allopolyploid is equivalent to both the mid-parent and the D-genome diploid. All three of the above, however, are differentially expressed relative to the A-genome diploid parent. The reverse situation is illustrated in lane 2, where expression in the D-genome parent is lower than that of the A-genome parent, rather than higher. Notice that in both lanes, expression in the allopolyploid mimics that of one parent, in this case the D genome, illustrating the general phenomenon of expression dominance of a single genome for both up- and down-regulated genes.

This observation of largely mid-parent expression masks an important underlying phenomenon, which we model in Figure 3. Specifically, for genes that differ in expression between the two parents, expression levels in the allopolyploid may be statistically equivalent both to one parent and the mid-parent, while these latter two values are statistically unequal themselves (Figure 3). Moreover, and importantly, all three of the foregoing values statistically differ from that of the other parent. The critical observation is that the dominant parent is recurrent for thousands of genes. Thus, allopolyploids display a strong expression bias, where expression is statistically equivalent to one of the two parents. This is true for both up-regulated and down-regulated genes. Hence, we term this phenomenon 'expression dominance'.

**Figure 4:**
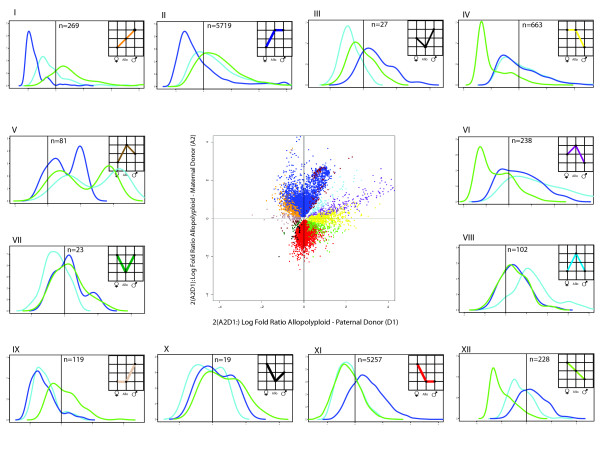
**Partitioning of expression patterns in allopolyploid *Gossypium*, A- and D-genome contrast**. Graphs inset into panels I to XII represent the possible expression patterns between two diploids and an allopolyploid derivative, where statistically differential expression is indicated by different vertical levels in each graph. Thus, for example, panel I in shows genes for which the paternal parent was differentially expressed and up-regulated relative to the allopolyploid, and the latter was up-regulated relative to the maternal parent. The total number of genes falling into each category is shown (*N*), as are the density distributions of expression levels (*y*-axis) for the genes involved in that particular expression pattern (maternal = dark blue; paternal = green; allopolyploid = light blue). In each of panels I to XII the *x*-axis represents standardized expression intensity. The average for the entire experiment is shown in each panel as the black horizontal line. All categories of gene expression are observed in both allopolyploids, although in remarkably different ratios (See Figure 5). Expression dominance of the paternal, *G*. *thurberi *genome in 2(A2D1) is shown by panels II and XI in Figure 4 (contrast with IV and IX), whereas the same phenomenon is shown (albeit to a lesser extent) for the *G. arboreum *genome in 2(A2G1) (same panels and contrast, Figure 5). In both cases, expression dominance reflects approximately equal amounts of up- and down-regulation to mimic the expression phenotype of the dominant parent. Transgressive up-regulation in each allopolyploid is partitioned into the possible constitutive categories and is shown by panels V, VI, and VIII; similarly, down-regulation is shown by panels III, VII, and X. The middle panel in each figure shows the distribution of expression divergence of each parent relative to the polyploid, with the maternal contrast on the *y *axis and the paternal contrast on the *x *axis, with colors corresponding to those used in the insets in panels I to XII.

**Figure 5:**
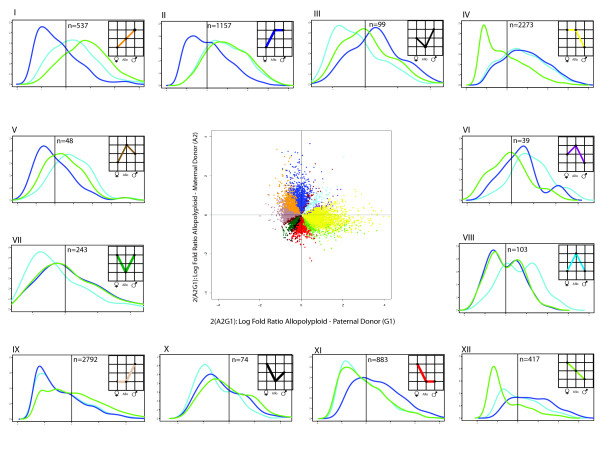
**Partitioning of expression patterns in allopolyploid *Gossypium*, A- and G-genome contrast**. Graphs inset into panels I to XII represent the possible expression patterns between two diploids and an allopolyploid derivative, where statistically differential expression is indicated by different vertical levels in each graph. The specific features of this figure and its relationship to Figure 4 are described in Figure legend 4.

To explore and categorize the expression alterations accompanying polyploid formation, we binned genes into the 12 possible patterns of differential expression for an allopolyploid and two parents (Figures 4 and 5) [24]. For genes differentially expressed between the parents, the most common result in both allopolyploids was the expression dominance of one parental expression phenotype, as described above, where expression dominance is operationally diagnosed as statistical equivalence of expression between the allopolyploid and its respective parent. Specifically, dominance of the paternal *G. thurberi *genome in the 2(A_2_D_1_) allopolyploid included nearly 11,000, or almost half, of all differentially expressed genes, with 52.1% being up-regulated to the paternal expression level and 47.9% down-regulated to the paternal expression level (panels II and XI in Figure 4A). Similarly, an expression dominance of a single parent exists in 2(A_2_G_1_), where about 5000 genes adopted the expression state of one parent, in this case *G. arboreum*, and again with approximately equal proportions (44% and 55%) of up- and down-regulation (Figures 4 and 5).

An additional dimension of this phenomenon is that expression dominance in the allopolyploid nucleus was reversed in the two systems; in 2(A_2_D_1_), the bulk of gene expression divergence was unequal to that from the maternal *G. arboreum *parent (or *G. arboreum *was 'expression recessive'), while this same parent displayed expression dominance in 2(A_2_G_1_). The magnitude of expression dominance was unequal in the two allopolyploids and was most extreme in the 2(A_2_D_1_) allopolyploid, where only 1769 genes were differentially expressed in leaves between the allopolyploid and the paternal parent, but eight times as many genes (13,863), representing fully a third of the genes on the chip, were differentially expressed in leaves between the allopolyploid and the maternal parent. These data constitute evidence for bidirectional, genome-wide expression dominance in allopolyploids, the direction of which may vary with the specific genomic combination involved.

The scope of expression dominance reported here is unprecedented, and suggests that the observation of mid-parent expression in allopolyploids (Figures 1 and 2) [15], while statistically correct, fails to capture the underlying dynamics of regulatory interactions that lead to genome-wide preferential expression of the phenotype contributed by one of the two genomes in an allopolyploid nucleus. In this light it seems likely that the statistical and analytical techniques used here would reveal that genomic dominance is more widespread than reported in *Arabidopsis*, where gene expression was studied in F_1 _hybrids between two allopolyploids [15]. Similar to our results for polyploid *Gossypium*, in *Arabidopsis *hybrids a relatively small percentage (5% to 6% in their case) of genes were differentially expressed in comparison to the mid-parent value. Of these, the general pattern observed was global repression (down-regulation) in the hybrid, with greater repression of genes that were up-regulated in *A. thaliana *(with respect to *A. arenosa*) than the reverse. Thus, for this small fraction of the total genes in the dataset that were studied (that is, only around the 5% that were differentially expressed from the mid-parent), there was differential expression repression in the hybrid with respect to the two parents. They did not explore the phenomenon of expression dominance for the greater than 95% of genes that were not differentially expressed from the mid-parent, an exploration that requires a categorically partitioned analysis of the full set of genes (cf. Figures 4 and 5). Thus, a newly discovered phenomenon associated with allopolyploidy is revealed in the present study, namely, global phenotypic expression dominance for both up- and down-regulated genes.

Although bidirectional expression dominance comprises the most common category of gene expression, all other expression possibilities were observed (Figures 4 and 5) in both allopolyploid systems studied, that is, 2(A_2_G_1_) and 2(A_2_D_1_). This includes transgressive up- and down-regulation in the allopolyploid, both for genes equivalently (Figures 4 and 5, panels VIII and VII, respectively, for up- and down-regulation) or differentially (Figures 4 and 5, panels V and VI, and III and X, respectively, for up- and down-regulation) expressed between the parents. Interestingly, although comparable numbers of genes exhibited statistically transgressive expression in the two allopolyploids (606 and 490 in 2(A_2_G_1_) and 2(A_2_D_1_), respectively), six times as many genes were up-regulated as down-regulated in 2(A_2_D_1_) (421 versus 69), whereas in 2(A_2_G_1_) the opposite trend was observed (190 up, 416 down). In addition, genes with novel down-regulation tended to have lower than average standardized expression, whereas, the converse is not true for genes with novel up-regulation (panels IV and IX in Figure 4).

It is tempting to speculate that genome-wide expression dominance and transgressive expression are connected to novel plant phenotypes and physiologies in allopolyploids. To explore this, we utilized Gene Ontology (GO) classifications for molecular and cellular function, coupled with Fisher's exact test, to identify processes over-represented in differentially expressed genes [25]. Between the genome-dominant parent and the allopolyploid, no significant terms emerge in either allopolyploid (Additional file 1), although we note that the non-genome-dominant parent largely shares the same differences from the allopolyploid as it does from the genome-dominant parent. Interestingly, genes transgressively transcribed in the allopolyploid are enriched for GO terms pertaining to cofactor binding, coenzyme binding, electron transport, oxioreductase activity, lyase activity, and the generation of precursor metabolites and energy, giving credence to the molecular underpinnings of the ecologically advantageous traits often seen in allopolyploids.

## Conclusion

Here we have shown that polyploidy in cotton is characterized by bidirectional genome-wide expression dominance, depending on the specific species combination and independent of the magnitude of gene expression. We also report massive expression divergence among diploid congeners in a common environment and the manner in which these differences become reconciled in a nascent polyploid. Our study illustrates the panoply of expression outcomes that duplicate genetic loci experience when divergent genomes become newly merged in a unified regulatory cellular milieu. At present the mechanistic underpinnings of expression dominance of a single genome in an allopolyploid remain elusive. Among the possibilities are asymmetries in the genomic distribution of methylation and other epigenetic marks, as suggested by recent work with *met1 *RNAi knockdowns in synthetic *Arabidopsis *polyploids, where expression differences in the allopolyploid were shown to be related to *de novo *changes in methylation [26]. Unlike *Arabidopsis*, methylation changes do not appear to accompany polyploidy in cotton [27], suggesting that the global expression dominance in this system is due to another mechanism, or likely, a suite of epigenetic mechanisms [28-31].

Notwithstanding our ignorance of the mechanism(s) responsible for expression dominance, this phenomenon, and indications of transgressive expression, provide clues into the enigma of the evolutionary success of allopolyploids. Future insights will derive from integrating shifts in gene expression into functional analyses in an ecologically relevant context, as well as from increased understanding of the molecular mechanisms by which they occur [14].

## Methods

### Plant material

Two synthetic allopolyploids and their diploid progenitors were used: (1) 2(A_2_G_1_) (Hyb-612 in Swanson-Wagner et al. [24]) was created by colchicine-doubling the hybrid between *G. arboreum *(accession no. 5265, as female) and *G. bickii *(accession no. 5048); (2) 2(A_2_D_1_) is a synthetic allopolyploid generated from the diploids *G. arboreum *(as female) and *G. thurberi *[33]. Ploidy level of the synthetic allopolyploids used has been confirmed by cytogenetic analysis [33-36]. These allopolyploids are largely phenotypically intermediate with respect to their diploid progenitors at the gross morphological level, although flower size is notably increased. The seeds used for this experiment were the C3 (post-colchicine doubling) generation for the 2(A_2_G_1_) material and fresh seed from a living, perpetually grown descendent of Beasley's original amphidiploid for the 2(A_2_D_1_). Seeds were scarified and germinated under high humidity in a 1:1 mix of sand and soil. Five plants of each taxon were grown in a randomized complete block design in each of three growth chambers in the Center for Plant Responses to Environmental Stresses in Bessey Hall, Iowa State University. Plants were grown at 26°C with 12-hour days, and watered as necessary. Plants were repotted into standard gallon containers after 3 weeks.

### RNA extraction and micro-array hybridization

The third through fifth fully expanded true leaves were harvested, divided into 1 g packets, flash-frozen in liquid N_2 _and stored at -80°C until extraction. RNA was extracted from five individuals from each of three growth chambers using a hot borate extraction/lithium chloride precipitation [37]. Equimolar amounts of high-quality RNA (assessed using a Bioanalyzer, Agilent, Santa Clara, CA, USA) were mixed from each of three individuals. Three replicates (corresponding to growth chambers) were hybridized to custom oligonucleotide micro-arrays using proprietary Nimblegen (Roche Nimblegen, Madison, WI, USA) protocols. These micro-arrays, described elsewhere [19,20], were designed using EST data from diploid A- and D-genome species as well as allopolyploid cotton [38], designed to minimize mismatch by selecting conserved sequence between species. Probes were designed from regions of overlap between the A- and D-genome species, where no sequence divergence had arisen. Additionally, whenever possible, probes were picked from regions of overlap with the AD-genome ESTs as well. The exonic sequence divergence between the A- and D-genome species < 1%, making the likelihood of broad-scale sequence mismatch on the chips unlikely [39]. Additionally, the G genome is more closely related to the A genome than D, suggesting that level of mismatch in our set of 60 mers is well below 1% [39]. The utility of these arrays and data validation using independent methods (quantitative real-time polymerase chain reaction and Sequenom mass array technologies) have been presented elsewhere [40,41].

### Statistical analysis

We employed standard micro-array data analysis techniques, as follows. Our Nimblegen platform assays each gene with a mean of seven unique probes per gene. To arrive at an estimate of expression for each unigene, we combined the probe values on a per-gene basis using Tukey's Biweight estimator [42]. In R software [43], the natural log of the intensity for each unigene was median-centered and scale-normalized. Estimates of gene expression were used to fit a linear model in SAS software taking the form:



where *y*_*ij *_is the normalized expression intensity of a unigene, *μ *is the intercept, δ_I _is the fixed effect of genotype *i*, with the random effect of replication *s*_*j*_, and the random error term *e*_*i*_. For each gene, we estimated the log-fold expression difference of four contrasts: the diploid parents to each other, each diploid parent to the allopolyploid and the mid-parent value to the allopolyploid. The mid-parent expression value was constructed in SAS software's PROC MIXED by down-weighting each parent in the contrast statement by 0.5; such an approach uses the pooled variance of both parental measures. The distribution of *P *values for each estimate was controlled for a false discovery rate using the method of Storey and Tibshirani [44] at a level of 0.05. Genes that were significantly differentially expressed were binned into classes using conditional statements, considering standardized expression levels and the statistical relationship between contrasts of interest. To explore the distribution of expression intensities among the 12 possible categories of expression patterns among two diploids and their derived allopolyploid, we mapped the kernel density of expression for each species using the density estimator in the R software package. These were plotted on a standardized scale against the experimental mean to illustrate inter-specific comparisons.

## Abbreviations

EST: expressed sequence tag; GO: Gene Ontology.

## Authors' contributions

JAU, RAR and JFW planned and designed the experiment, JAU and RAR planted, grew and harvested the biological material and prepped the RNA. RAR conducted the micro-array experiments and analyzed the data. RAR drafted the manuscript with substantial input from JFW and JAU.

## Supplementary Material

Additional file 1 **Table S1**. Blast2GO annotation for the genes differentially expressed in this experiment. Genes are binned by contrast, with the contrast specified as the header. All possible contrasts are reported (AvG, AvAG, GvAG, DvA, DvAD, AvAD and those genes from the transgressive category). Click here for file
