# Supplementary material for: Genomic expression dominance in allopolyploids
Source: BMC Biol. 2009 May 1;7:18. doi: 10.1186/1741-7007-7-18 (PMC2684529; doi:10.1186/1741-7007-7-18)
Supplement: Additional file 1 — Table S1. Blast2GO annotation for the genes differentially expressed in this experiment. Genes are binned by contrast, with the contrast specified as the header. All possible contrasts are reported (AvG, AvAG, GvAG, DvA, DvAD, AvAD and those genes from the transgressive category). [file 1741-7007-7-18-S1.pdf]

Tests for all terms in Gene Ontology whether it is enriched in a test group when compared to a reference group using Fisher's excact test with Multiple Testing.
Pub: Biological Profiling of Gene Groups utilizing Gene Ontology A Statistical Framework
Poster: GOSSIP: Biological Profiling of Gene Groups utilizing Gene Ontology
by Nils Blthgen, Karsten Brand, Hanspeter Herzel, Dieter Beule

GO Term	Name	FDR	FWER	single test p-Value	# in test group	# in reference group	# non annot test	# non annot reference group	Over/Under
GO:0048037	cofactor binding	2.3179E-6	5.79474E-7	1.83507E-9	207	768	5872	35688	over
GO:0016829	lyase activity	2.62833E-6	1.31416E-6	4.0753E-9	246	964	5833	35492	over
GO:0006091	generation of precursor metabolites and energy	7.49382E-5	5.6202E-5	1.63317E-7	418	1900	5661	34556	over
GO:0003824	catalytic activity	3.03254E-4	3.16145E-4	8.90882E-7	2817	15692	3262	20764	over
GO:0050662	coenzyme binding	3.03254E-4	3.78995E-4	1.06727E-6	150	571	5929	35885	over
GO:0044237	cellular metabolic process	3.21396E-4	4.81978E-4	1.22996E-6	2666	14811	3413	21645	over
GO:0019752	carboxylic acid metabolic process	5.4923E-4	0.00119199	3.20212E-6	380	1763	5699	34693	over
GO:0008152	metabolic process	5.4923E-4	0.00121384	3.28311E-6	3177	17911	2902	18545	over
GO:0006082	organic acid metabolic process	5.4923E-4	0.001235	3.34865E-6	380	1764	5699	34692	over
GO:0009628	response to abiotic stimulus	6.39254E-4	0.00159686	4.32665E-6	405	1903	5674	34553	over
GO:0016832	aldehyde-lyase activity	8.02119E-4	0.00244903	5.97685E-6	26	48	6053	36408	over
GO:0006950	response to stress	8.02119E-4	0.00274807	7.02525E-6	466	2243	5613	34213	over
GO:0016837	carbon-oxygen lyase activity, acting on polysaccharides	8.02119E-4	0.00280348	7.18349E-6	23	39	6056	36417	over
GO:0030570	pectate lyase activity	8.02119E-4	0.00280348	7.18349E-6	23	39	6056	36417	over
GO:0015979	photosynthesis	0.00154513	0.0057775	1.33812E-5	138	545	5941	35911	over
GO:0046483	heterocycle metabolic process	0.00174583	0.006959	1.71487E-5	92	329	5987	36127	over
GO:0006118	electron transport	0.00218055	0.00922454	2.36357E-5	287	1315	5792	35141	over
GO:0016835	carbon-oxygen lyase activity	0.00333071	0.0148765	3.6329E-5	86	309	5993	36147	over
GO:0030170	pyridoxal phosphate binding	0.00337485	0.0159028	3.99006E-5	52	158	6027	36298	over
GO:0051186	cofactor metabolic process	0.00385112	0.0190715	4.86127E-5	191	830	5888	35626	over
GO:0010287	plastoglobule	0.00410514	0.0213215	5.11269E-5	10	8	6069	36448	over
GO:0044248	cellular catabolic process	0.00645882	0.0355738	8.29548E-5	317	1503	5762	34953	over
GO:0009308	amine metabolic process	0.00645882	0.0378434	8.93988E-5	244	1117	5835	35339	over
GO:0016830	carbon-carbon lyase activity	0.00645882	0.0380119	8.95619E-5	102	395	5977	36061	over
GO:0006520	amino acid metabolic process	0.0067899	0.0439848	9.94541E-5	218	983	5861	35473	over
GO:0004332	fructose-bisphosphate aldolase activity	0.0067899	0.044666	1.01449E-4	19	35	6060	36421	over
GO:0009056	catabolic process	0.0067899	0.0447978	1.02282E-4	322	1535	5757	34921	over
GO:0044435	plastid part	0.00811432	0.0563597	1.26559E-4	167	724	5912	35732	over
GO:0044238	primary metabolic process	0.00811432	0.0575927	1.31701E-4	2634	14884	3445	21572	over
GO:0005975	carbohydrate metabolic process	0.00811432	0.0590432	1.37577E-4	401	1971	5678	34485	over
GO:0009507	chloroplast	0.00812242	0.0610091	1.44984E-4	633	3257	5446	33199	over
GO:0044434	chloroplast part	0.00968874	0.0745832	1.82802E-4	154	664	5925	35792	over
GO:0009532	plastid stroma	0.00991125	0.0806287	1.9077E-4	73	266	6006	36190	over
GO:0006096	glycolysis	0.00991125	0.0830083	2.00028E-4	86	328	5993	36128	over
GO:0042803	protein homodimerization activity	0.00991125	0.0833571	2.0134E-4	22	48	6057	36408	over
GO:0009570	chloroplast stroma	0.00991125	0.0853399	2.03413E-4	64	225	6015	36231	over
GO:0033013	tetrapyrrole metabolic process	0.0138247	0.123079	3.13947E-4	43	135	6036	36321	over
GO:0006778	porphyrin metabolic process	0.0138247	0.123079	3.13947E-4	43	135	6036	36321	over
GO:0006519	amino acid and derivative metabolic process	0.0153643	0.143544	3.35663E-4	290	1393	5789	35063	over
GO:0050660	FAD binding	0.0153643	0.144647	3.41326E-4	49	162	6030	36294	over
GO:0006779	porphyrin biosynthetic process	0.0153643	0.148989	3.50762E-4	36	106	6043	36350	over
GO:0033014	tetrapyrrole biosynthetic process	0.0153643	0.148989	3.50762E-4	36	106	6043	36350	over
GO:0006807	nitrogen compound metabolic process	0.0207929	0.200311	4.38613E-4	256	1217	5823	35239	over
GO:0004240	mitochondrial processing peptidase activity	0.022799	0.222402	4.8339E-4	6	3	6073	36453	over
GO:0043170	macromolecule metabolic process	0.022799	0.226245	4.908E-4	2002	11229	4077	25227	over
GO:0044262	cellular carbohydrate metabolic process	0.0252809	0.252293	5.51227E-4	216	1009	5863	35447	over
GO:0016620	oxidoreductase activity, acting on the aldehyde or oxo group of donors, NAD or NADP as acceptor	0.0262154	0.268653	6.27108E-4	44	145	6035	36311	over
GO:0009408	response to heat	0.0262154	0.26992	6.33421E-4	71	270	6008	36186	over
GO:0015297	antiporter activity	0.0286598	0.299185	7.42643E-4	43	142	6036	36314	over

GO:0009987	cellular process	0.0286598	0.301115	7.55794E-4	3304	19011	2775	17445	over
GO:0015976	carbon utilization	0.0301408	0.323045	8.379E-4	57	207	6022	36249	over
GO:0006563	L-serine metabolic process	0.0301408	0.325532	8.54206E-4	30	87	6049	36369	over
GO:0015995	chlorophyll biosynthetic process	0.0301408	0.329273	8.73069E-4	29	83	6050	36373	over
GO:0050896	response to stimulus	0.0309105	0.341192	9.26069E-4	904	4874	5175	31582	over
GO:0005222	intracellular cAMP activated cation channel activity	0.0385107	0.426262	0.00106187	6	4	6073	36452	over
GO:0005221	intracellular cyclic nucleotide activated cation channel activity	0.0385107	0.426262	0.00106187	6	4	6073	36452	over
GO:0043855	cyclic nucleotide-gated ion channel activity	0.0385107	0.426262	0.00106187	6	4	6073	36452	over
GO:0009314	response to radiation	0.0385107	0.43264	0.00109927	170	781	5909	35675	over
GO:0044265	cellular macromolecule catabolic process	0.0385107	0.433392	0.00110675	200	940	5879	35516	over
GO:0016491	oxidoreductase activity	0.0388396	0.444612	0.00116056	617	3249	5462	33207	over
GO:0016052	carbohydrate catabolic process	0.0388396	0.452326	0.00123692	117	508	5962	35948	over
GO:0044275	cellular carbohydrate catabolic process	0.0388396	0.452326	0.00123692	117	508	5962	35948	over
GO:0015994	chlorophyll metabolic process	0.043687	0.510384	0.00147503	34	108	6045	36348	over
GO:0042651	thylakoid membrane	0.043687	0.5153	0.00151115	69	271	6010	36185	over
GO:0006766	vitamin metabolic process	0.043687	0.520635	0.00155536	65	252	6014	36204	over
GO:0046365	monosaccharide catabolic process	0.043687	0.524208	0.00161816	100	426	5979	36030	over
GO:0006007	glucose catabolic process	0.043687	0.524208	0.00161816	100	426	5979	36030	over
GO:0019320	hexose catabolic process	0.043687	0.524208	0.00161816	100	426	5979	36030	over
GO:0009416	response to light stimulus	0.0448405	0.539149	0.00165477	167	774	5912	35682	over
GO:0004930	G-protein coupled receptor activity	0.0448405	0.543796	0.001658	13	25	6066	36431	over
GO:0019685	photosynthesis, dark reaction	0.0474148	0.56904	0.00183696	39	132	6040	36324	over

AvG

Tests for all terms in Gene Ontology whether it is enriched in a test group when compared to a reference group using Fisher's excact test with Multiple Testing.
Pub: Biological Profiling of Gene Groups utilizing Gene Ontology A Statistical Framework
Poster: GOSSIP: Biological Profiling of Gene Groups utilizing Gene Ontology
by Nils Blthgen, Karsten Brand, Hanspeter Herzel, Dieter Beule

GO Term	Name	FDR	FWER	single test p-Value	# in test group	# in reference group	# non annot test	# non annot reference group	Over/Under
GO:0016829	lyase activity	4.92268E-6	1.23067E-6	4.11103E-9	255	955	6100	35225	over
GO:0048037	cofactor binding	1.70125E-5	8.50623E-6	2.6362E-8	209	766	6146	35414	over
GO:0009507	chloroplast	1.41118E-4	1.05833E-4	2.7724E-7	690	3200	5665	32980	over
GO:0015976	carbon utilization	2.64284E-4	2.64249E-4	7.40276E-7	70	194	6285	35986	over
GO:0006091	generation of precursor metabolites and energy	2.82823E-4	3.94527E-4	1.08833E-6	428	1890	5927	34290	over
GO:0009628	response to abiotic stimulus	2.82823E-4	4.49898E-4	1.19917E-6	426	1882	5929	34298	over
GO:0015979	photosynthesis	2.82823E-4	5.14407E-4	1.42226E-6	148	535	6207	35645	over
GO:0009570	chloroplast stroma	2.82823E-4	5.65486E-4	1.52741E-6	74	215	6281	35965	over
GO:0009532	plastid stroma	4.55882E-4	0.00102521	2.5714E-6	83	256	6272	35924	over
GO:0016830	carbon-carbon lyase activity	5.90914E-4	0.0014762	4.03414E-6	112	385	6243	35795	over
GO:0006096	glycolysis	7.21532E-4	0.00215543	5.50852E-6	96	318	6259	35862	over
GO:0015977	carbon utilization by fixation of carbon dioxide	7.21532E-4	0.00221908	5.68723E-6	61	172	6294	36008	over
GO:0044237	cellular metabolic process	7.21532E-4	0.00234223	6.28927E-6	2770	14707	3585	21473	over
GO:0009056	catabolic process	7.38547E-4	0.00260144	7.05E-6	345	1512	6010	34668	over
GO:0044435	plastid part	7.38547E-4	0.00276572	7.68195E-6	181	710	6174	35470	over
GO:0050662	coenzyme binding	7.82713E-4	0.00312596	8.07906E-6	151	570	6204	35610	over
GO:0044434	chloroplast part	8.00709E-4	0.00339723	8.44156E-6	168	650	6187	35530	over
GO:0010287	plastoglobule	8.32732E-4	0.00380208	9.37959E-6	11	7	6344	36173	over
GO:0019752	carboxylic acid metabolic process	8.32732E-4	0.0040412	9.98534E-6	391	1752	5964	34428	over
GO:0006082	organic acid metabolic process	8.32732E-4	0.00415501	1.0429E-5	391	1753	5964	34427	over
GO:0044248	cellular catabolic process	0.00100786	0.00544782	1.1964E-5	337	1483	6018	34697	over
GO:0008152	metabolic process	0.00100786	0.00560634	1.26647E-5	3306	17782	3049	18398	over
GO:0016832	aldehyde-lyase activity	0.00100786	0.00577845	1.34773E-5	26	48	6329	36132	over
GO:0006950	response to stress	0.00102075	0.00610582	1.4256E-5	482	2227	5873	33953	over
GO:0019685	photosynthesis, dark reaction	0.00114892	0.00715501	1.6622E-5	47	124	6308	36056	over
GO:0015995	chlorophyll biosynthetic process	0.00154715	0.0102513	2.60355E-5	34	78	6321	36102	over
GO:0006118	electron transport	0.00154715	0.010389	2.65557E-5	298	1304	6057	34876	over

GO:0030170	pyridoxal phosphate binding	0.00183094	0.0127348	3.23647E-5	54	156	6301	36024	over
GO:0003824	catalytic activity	0.00226495	0.0162869	3.92692E-5	2910	15599	3445	20581	over
GO:0004332	fructose-bisphosphate aldolase activity	0.00296822	0.0220158	5.49865E-5	20	34	6335	36146	over
GO:0019253	reductive pentose-phosphate cycle	0.00327275	0.025045	6.58538E-5	43	117	6312	36063	over
GO:0015994	chlorophyll metabolic process	0.00519053	0.0406742	8.45316E-5	39	103	6316	36077	over
GO:0006778	porphyrin metabolic process	0.00558981	0.0464027	1.04698E-4	46	132	6309	36048	over
GO:0033013	tetrapyrrole metabolic process	0.00558981	0.0464027	1.04698E-4	46	132	6309	36048	over
GO:0005221	intracellular cyclic nucleotide activated cation channel activity	0.00626245	0.0562824	1.30815E-4	7	3	6348	36177	over
GO:0043855	cyclic nucleotide-gated ion channel activity	0.00626245	0.0562824	1.30815E-4	7	3	6348	36177	over
GO:0005222	intracellular cAMP activated cation channel activity	0.00626245	0.0562824	1.30815E-4	7	3	6348	36177	over
GO:0051186	cofactor metabolic process	0.00689635	0.063416	1.50146E-4	195	826	6160	35354	over
GO:0016835	carbon-oxygen lyase activity	0.00790203	0.0744764	1.72876E-4	86	309	6269	35871	over
GO:0033014	tetrapyrrole biosynthetic process	0.00790203	0.0793188	1.85985E-4	38	104	6317	36076	over
GO:0006779	porphyrin biosynthetic process	0.00790203	0.0793188	1.85985E-4	38	104	6317	36076	over
GO:0009266	response to temperature stimulus	0.00790203	0.0796236	1.88406E-4	159	654	6196	35526	over
GO:0042440	pigment metabolic process	0.008104	0.0893582	2.18026E-4	65	218	6290	35962	over
GO:0044238	primary metabolic process	0.008104	0.0901252	2.24755E-4	2745	14773	3610	21407	over
GO:0042548	regulation of photosynthesis, light reaction	0.008104	0.0908305	2.25539E-4	20	39	6335	36141	over
GO:0010109	regulation of photosynthesis	0.008104	0.0908305	2.25539E-4	20	39	6335	36141	over
GO:0043467	regulation of generation of precursor metabolites and energy	0.008104	0.0908305	2.25539E-4	20	39	6335	36141	over
GO:0006520	amino acid metabolic process	0.0108138	0.121701	2.88515E-4	223	978	6132	35202	over
GO:0046365	monosaccharide catabolic process	0.0112709	0.133859	3.15114E-4	108	418	6247	35762	over
GO:0006007	glucose catabolic process	0.0112709	0.133859	3.15114E-4	108	418	6247	35762	over
GO:0019320	hexose catabolic process	0.0112709	0.133859	3.15114E-4	108	418	6247	35762	over
GO:0009308	amine metabolic process	0.0116873	0.140959	3.31713E-4	249	1112	6106	35068	over
GO:0046483	heterocycle metabolic process	0.0124921	0.152552	3.80855E-4	89	332	6266	35848	over
GO:0006519	amino acid and derivative metabolic process	0.0141745	0.179793	4.06254E-4	301	1382	6054	34798	over
GO:0019842	vitamin binding	0.0141745	0.181311	4.12738E-4	73	260	6282	35920	over
GO:0006006	glucose metabolic process	0.0141745	0.184041	4.30197E-4	113	446	6242	35734	over
GO:0051287	NAD binding	0.0141745	0.184122	4.30856E-4	52	168	6303	36012	over
GO:0005975	carbohydrate metabolic process	0.0141745	0.185791	4.41888E-4	412	1960	5943	34220	over
GO:0042651	thylakoid membrane	0.0143904	0.191251	4.72139E-4	74	266	6281	35914	over
GO:0016052	carbohydrate catabolic process	0.0168851	0.227025	5.07081E-4	124	501	6231	35679	over
GO:0044275	cellular carbohydrate catabolic process	0.0168851	0.227025	5.07081E-4	124	501	6231	35679	over
GO:0046164	alcohol catabolic process	0.017954	0.242931	5.56491E-4	108	426	6247	35754	over
GO:0044265	cellular macromolecule catabolic process	0.0211144	0.282922	6.49568E-4	210	930	6145	35250	over
GO:0051188	cofactor biosynthetic process	0.0225688	0.303106	6.94513E-4	109	434	6246	35746	over
GO:0044255	cellular lipid metabolic process	0.0228087	0.309724	7.13599E-4	227	1018	6128	35162	over
GO:0009987	cellular process	0.0253358	0.344888	8.23923E-4	3450	18865	2905	17315	over
GO:0050896	response to stimulus	0.0253358	0.357154	9.14976E-4	943	4835	5412	31345	over
GO:0050660	FAD binding	0.0253358	0.360417	9.35278E-4	49	162	6306	36018	over
GO:0006807	nitrogen compound metabolic process	0.0253358	0.373203	0.00100279	263	1210	6092	34970	over
GO:0043481	anthocyanin accumulation in tissues in response to UV light	0.0253358	0.378168	0.00100973	14	25	6341	36155	over
GO:0043473	pigmentation	0.0253358	0.378168	0.00100973	14	25	6341	36155	over
GO:0043480	pigment accumulation in tissues	0.0253358	0.378168	0.00100973	14	25	6341	36155	over
GO:0043478	pigment accumulation in response to UV light	0.0253358	0.378168	0.00100973	14	25	6341	36155	over
GO:0043479	pigment accumulation in tissues in response to UV light	0.0253358	0.378168	0.00100973	14	25	6341	36155	over
GO:0043476	pigment accumulation	0.0253358	0.378168	0.00100973	14	25	6341	36155	over
GO:0009747	hexokinase-dependent signaling	0.030946	0.447258	0.00119763	5	2	6350	36178	over
GO:0006629	lipid metabolic process	0.030946	0.448863	0.00122276	255	1174	6100	35006	over
GO:0009853	photorespiration	0.0321957	0.46628	0.00125715	40	126	6315	36054	over
GO:0016837	carbon-oxygen lyase activity, acting on polysaccharides	0.0329061	0.482215	0.00130477	19	43	6336	36137	over
GO:0030570	pectate lyase activity	0.0329061	0.482215	0.00130477	19	43	6336	36137	over
GO:0009314	response to radiation	0.0341651	0.50573	0.00136835	176	775	6179	35405	over
GO:0009414	response to water deprivation	0.0341651	0.507763	0.00140163	86	335	6269	35845	over

GO:0005996	monosaccharide metabolic process	0.0341651	0.507868	0.0014036	144	616	6211	35564	over
GO:0044262	cellular carbohydrate metabolic process	0.0344623	0.519066	0.00144051	221	1004	6134	35176	over
GO:0006066	alcohol metabolic process	0.0344623	0.519252	0.00144283	168	736	6187	35444	over
GO:0009063	amino acid catabolic process	0.0347659	0.535293	0.00149231	48	162	6307	36018	over
GO:0009310	amine catabolic process	0.0347659	0.535293	0.00149231	48	162	6307	36018	over
GO:0019252	starch biosynthetic process	0.0347659	0.537803	0.00150531	27	74	6328	36106	over
GO:0009416	response to light stimulus	0.0347659	0.53867	0.00150711	174	767	6181	35413	over
GO:0046148	pigment biosynthetic process	0.0356031	0.5512	0.00163321	53	185	6302	35995	over
GO:0006563	L-serine metabolic process	0.0370657	0.56974	0.00174767	30	87	6325	36093	over
GO:0010205	photoinhibition	0.0383944	0.590495	0.00189713	13	24	6342	36156	over
GO:0043155	negative regulation of photosynthesis, light reaction	0.0383944	0.590495	0.00189713	13	24	6342	36156	over
GO:0019318	hexose metabolic process	0.0395582	0.605658	0.00197218	133	568	6222	35612	over
GO:0009415	response to water	0.0395582	0.60924	0.00199288	90	359	6265	35821	over
GO:0016491	oxidoreductase activity	0.0438079	0.650616	0.0021865	639	3227	5716	32953	over
GO:0032787	monocarboxylic acid metabolic process	0.0491449	0.696388	0.00235005	156	686	6199	35494	over

GvAG

Tests for all terms in Gene Ontology whether it is enriched in a test group when compared to a reference group using Fisher's excact test with Multiple Testing.
Pub: Biological Profiling of Gene Groups utilizing Gene Ontology A Statistical Framework
Poster: GOSSIP: Biological Profiling of Gene Groups utilizing Gene Ontology
by Nils Blthgen, Karsten Brand, Hanspeter Herzel, Dieter Beule

GO Term	Name	FDR	FWER	single test p-Value	# in test group	# in reference group	# non annot test	# non annot reference group	Over/Under
GO:0048037	cofactor binding	9.14235E-5	2.28556E-5	6.75373E-8	183	792	5276	36284	over
GO:0016829	lyase activity	4.93803E-4	3.23911E-4	8.81E-7	213	997	5246	36079	over
GO:0006091	generation of precursor metabolites and energy	4.93803E-4	4.43355E-4	1.23131E-6	374	1944	5085	35132	over
GO:0019752	carboxylic acid metabolic process	4.93803E-4	5.96997E-4	1.60525E-6	348	1795	5111	35281	over
GO:0006082	organic acid metabolic process	4.93803E-4	6.17063E-4	1.67644E-6	348	1796	5111	35280	over
GO:0009308	amine metabolic process	5.96716E-4	8.94674E-4	2.29663E-6	233	1128	5226	35948	over
GO:0006520	amino acid metabolic process	0.00172976	0.0030225	7.66834E-6	206	995	5253	36081	over
GO:0006118	electron transport	0.00203641	0.00406453	9.96148E-6	264	1338	5195	35738	over
GO:0003824	catalytic activity	0.00208035	0.00468824	1.14973E-5	2521	15988	2938	21088	over
GO:0009628	response to abiotic stimulus	0.00208035	0.00529527	1.37305E-5	364	1944	5095	35132	over
GO:0050662	coenzyme binding	0.00208035	0.00570464	1.52255E-5	132	589	5327	36487	over
GO:0008152	metabolic process	0.00243338	0.00727358	1.90606E-5	2849	18239	2610	18837	over
GO:0006807	nitrogen compound metabolic process	0.00245526	0.00794787	2.06239E-5	243	1230	5216	35846	over
GO:0006519	amino acid and derivative metabolic process	0.00282536	0.00984004	2.29834E-5	273	1410	5186	35666	over
GO:0006950	response to stress	0.00337883	0.0133637	3.25844E-5	417	2292	5042	34784	over
GO:0016832	aldehyde-lyase activity	0.00337883	0.0134244	3.28589E-5	23	51	5436	37025	over
GO:0004332	fructose-bisphosphate aldolase activity	0.00784208	0.0347935	8.35163E-5	18	36	5441	37040	over
GO:0044237	cellular metabolic process	0.00784208	0.0364332	8.90294E-5	2371	15106	3088	21970	over
GO:0030170	pyridoxal phosphate binding	0.00784208	0.0365649	8.95704E-5	47	163	5412	36913	over
GO:0009507	chloroplast	0.0232305	0.109663	2.5237E-4	570	3320	4889	33756	over
GO:0043467	regulation of generation of precursor metabolites and energy	0.0272185	0.144877	2.95996E-4	18	41	5441	37035	over
GO:0042548	regulation of photosynthesis, light reaction	0.0272185	0.144877	2.95996E-4	18	41	5441	37035	over
GO:0010109	regulation of photosynthesis	0.0272185	0.144877	2.95996E-4	18	41	5441	37035	over
GO:0006537	glutamate biosynthetic process	0.0334378	0.181788	4.13828E-4	9	11	5450	37065	over
GO:0016835	carbon-oxygen lyase activity	0.0385222	0.214608	5.18672E-4	74	321	5385	36755	over
GO:0016837	carbon-oxygen lyase activity, acting on polysaccharides	0.0385222	0.228977	5.78439E-4	18	44	5441	37032	over
GO:0030570	pectate lyase activity	0.0385222	0.228977	5.78439E-4	18	44	5441	37032	over
GO:0009532	plastid stroma	0.0389146	0.244492	5.82694E-4	65	274	5394	36802	over
GO:0044238	primary metabolic process	0.0389146	0.256787	5.9628E-4	2359	15159	3100	21917	over
GO:0009570	chloroplast stroma	0.0389146	0.260949	6.03066E-4	57	232	5402	36844	over
GO:0000139	Golgi membrane	0.0389146	0.265571	6.10293E-4	22	61	5437	37015	over
GO:0005975	carbohydrate metabolic process	0.0389146	0.269469	6.26581E-4	357	2015	5102	35061	over
GO:0006790	sulfur metabolic process	0.0389146	0.280173	6.58237E-4	57	233	5402	36843	over
GO:0009056	catabolic process	0.0389146	0.283466	6.77811E-4	285	1572	5174	35504	over

GO:0048583	regulation of response to stimulus	0.0389146	0.296034	7.2161E-4	17	41	5442	37035	over
GO:0015995	chlorophyll biosynthetic process	0.0389146	0.308743	7.99916E-4	27	85	5432	36991	over
GO:0030658	transport vesicle membrane	0.0389146	0.315757	8.03005E-4	11	19	5448	37057	over
GO:0030660	Golgi-associated vesicle membrane	0.0389146	0.315757	8.03005E-4	11	19	5448	37057	over
GO:0012510	trans-Golgi network transport vesicle membrane	0.0389146	0.315757	8.03005E-4	11	19	5448	37057	over
GO:0030130	clathrin coat of trans-Golgi network vesicle	0.0435135	0.366649	9.63295E-4	9	13	5450	37063	over
GO:0010287	plastoglobule	0.0435135	0.373876	9.65853E-4	8	10	5451	37066	over
GO:0015979	photosynthesis	0.0435135	0.374961	9.72119E-4	116	567	5343	36509	over
GO:0043284	biopolymer biosynthetic process	0.0435135	0.379283	0.00100682	103	493	5356	36583	over
GO:0009066	aspartate family amino acid metabolic process	0.0435135	0.382362	0.00102329	53	217	5406	36859	over
GO:0051186	cofactor metabolic process	0.0435135	0.387109	0.00103872	165	856	5294	36220	over

Transgressive

GOSSIP
Tests for all terms in Gene Ontology whether it is enriched in a test group when compared to a reference group using Fisher's excact test with Multiple Testing.
Pub: Biological Profiling of Gene Groups utilizing Gene Ontology A Statistical Framework
Poster: GOSSIP: Biological Profiling of Gene Groups utilizing Gene Ontology
by Nils Blthgen, Karsten Brand, Hanspeter Herzel, Dieter Beule

GO Term	Name	FDR	FWER	single test p-Value	# in test group	# in reference group	# non annot test	# non annot reference group	Over/Under
GO:0048037	cofactor binding	5.63795E-4	1.40939E-4	4.58576E-7	54	921	1078	40482	over
GO:0050662	coenzyme binding	0.00922971	0.00460423	1.32804E-5	40	681	1092	40722	over
GO:0006118	electron transport	0.0197182	0.0149881	4.10567E-5	70	1532	1062	39871	over
GO:0016491	oxidoreductase activity	0.0197182	0.0195252	5.49484E-5	142	3724	990	37679	over
GO:0016829	lyase activity	0.0327771	0.0401437	1.00114E-4	55	1155	1077	40248	over
GO:0006091	generation of precursor metabolites and energy	0.0401724	0.0584796	1.52302E-4	91	2227	1041	39176	over

AvD

Tests for all terms in Gene Ontology whether it is enriched in a test group when compared to a reference group using Fisher's excact test with Multiple Testing.
Pub: Biological Profiling of Gene Groups utilizing Gene Ontology A Statistical Framework
Poster: GOSSIP: Biological Profiling of Gene Groups utilizing Gene Ontology
by Nils Blthgen, Karsten Brand, Hanspeter Herzel, Dieter Beule

GO Term	Name	FDR	FWER	single test p-Value	# in test group	# in reference group	# non annot test	# non annot reference group	Over/Under
GO:0016829	lyase activity	1.29319E-6	3.23297E-7	9.90259E-10	269	941	6417	34908	over
GO:0048037	cofactor binding	2.35458E-6	1.17729E-6	3.57739E-9	222	753	6464	35096	over
GO:0006091	generation of precursor metabolites and energy	4.90566E-6	3.67924E-6	1.14387E-8	463	1855	6223	33994	over
GO:0050662	coenzyme binding	1.33985E-4	1.50016E-4	4.07165E-7	164	557	6522	35292	over
GO:0019752	carboxylic acid metabolic process	1.33985E-4	1.82127E-4	4.87881E-7	420	1723	6266	34126	over
GO:0006082	organic acid metabolic process	1.33985E-4	2.03113E-4	5.13474E-7	420	1724	6266	34125	over
GO:0009628	response to abiotic stimulus	1.33985E-4	2.34447E-4	6.09351E-7	448	1860	6238	33989	over
GO:0006118	electron transport	0.00104648	0.00209078	5.48119E-6	317	1285	6369	34564	over
GO:0003824	catalytic activity	0.00141278	0.00376743	9.82172E-6	3069	15440	3617	20409	over
GO:0030570	pectate lyase activity	0.00141278	0.0038776	1.02744E-5	24	38	6662	35811	over
GO:0016837	carbon-oxygen lyase activity, acting on polysaccharides	0.00141278	0.0038776	1.02744E-5	24	38	6662	35811	over
GO:0006950	response to stress	0.00169086	0.00527298	1.30697E-5	505	2204	6181	33645	over
GO:0015979	photosynthesis	0.00169086	0.00587612	1.45583E-5	149	534	6537	35315	over
GO:0008152	metabolic process	0.00169086	0.00590054	1.48543E-5	3472	17616	3214	18233	over
GO:0016835	carbon-oxygen lyase activity	0.002224	0.00830532	1.78495E-5	94	301	6592	35548	over
GO:0016830	carbon-carbon lyase activity	0.00241475	0.0103528	2.41606E-5	113	384	6573	35465	over
GO:0044237	cellular metabolic process	0.00241475	0.0103691	2.43046E-5	2898	14579	3788	21270	over
GO:0009507	chloroplast	0.00241475	0.0108076	2.55532E-5	701	3189	5985	32660	over
GO:0015976	carbon utilization	0.00306351	0.0144463	3.37497E-5	67	197	6619	35652	over
GO:0006096	glycolysis	0.00354918	0.0175894	4.30167E-5	96	318	6590	35531	over
GO:0009056	catabolic process	0.00436928	0.0226777	5.66265E-5	353	1504	6333	34345	over
GO:0044248	cellular catabolic process	0.00521616	0.0287752	6.63439E-5	346	1474	6340	34375	over
GO:0006520	amino acid metabolic process	0.00521616	0.0295477	7.04163E-5	238	963	6448	34886	over
GO:0009308	amine metabolic process	0.00731095	0.0443535	9.65193E-5	265	1096	6421	34753	over
GO:0016832	aldehyde-lyase activity	0.00731095	0.0446656	9.79623E-5	25	49	6661	35800	over
GO:0009570	chloroplast stroma	0.00810778	0.0513364	1.10456E-4	70	219	6616	35630	over

GO:0030170	pyridoxal phosphate binding	0.00923482	0.0604326	1.27889E-4	54	156	6632	35693	over
GO:0046483	heterocycle metabolic process	0.00928837	0.0629506	1.35847E-4	95	326	6591	35523	over
GO:0009532	plastid stroma	0.010164	0.0710401	1.63157E-4	79	260	6607	35589	over
GO:0016491	oxidoreductase activity	0.0129929	0.092851	2.22419E-4	685	3181	6001	32668	over
GO:0050896	response to stimulus	0.0145404	0.1097	2.55662E-4	999	4779	5687	31070	over
GO:0019685	photosynthesis, dark reaction	0.0145404	0.109815	2.55713E-4	45	126	6641	35723	over
GO:0050660	FAD binding	0.0148231	0.115112	2.75337E-4	53	158	6633	35691	over
GO:0009314	response to radiation	0.0155255	0.134103	3.18105E-4	189	762	6497	35087	over
GO:0006519	amino acid and derivative metabolic process	0.0155255	0.135556	3.26602E-4	316	1367	6370	34482	over
GO:0006778	porphyrin metabolic process	0.0155255	0.139605	3.42272E-4	46	132	6640	35717	over
GO:0033013	tetrapyrrole metabolic process	0.0155255	0.139605	3.42272E-4	46	132	6640	35717	over
GO:0015977	carbon utilization by fixation of carbon dioxide	0.0155255	0.139909	3.45571E-4	57	176	6629	35673	over
GO:0051186	cofactor metabolic process	0.0155255	0.141909	3.5215E-4	201	820	6485	35029	over
GO:0044435	plastid part	0.0155255	0.145499	3.59329E-4	178	713	6508	35136	over
GO:0044434	chloroplast part	0.0155255	0.147124	3.63631E-4	165	653	6521	35196	over
GO:0015995	chlorophyll biosynthetic process	0.017069	0.164087	4.04844E-4	32	80	6654	35769	over
GO:0044265	cellular macromolecule catabolic process	0.0186682	0.181834	4.47479E-4	221	919	6465	34930	over
GO:0009416	response to light stimulus	0.0193655	0.191867	4.65593E-4	186	755	6500	35094	over
GO:0006779	porphyrin biosynthetic process	0.0218358	0.229949	5.20039E-4	38	104	6648	35745	over
GO:0033014	tetrapyrrole biosynthetic process	0.0218358	0.229949	5.20039E-4	38	104	6648	35745	over
GO:0042440	pigment metabolic process	0.0218358	0.230178	5.21194E-4	66	217	6620	35632	over
GO:0006807	nitrogen compound metabolic process	0.0218358	0.23104	5.25923E-4	278	1195	6408	34654	over
GO:0044238	primary metabolic process	0.0218358	0.234711	5.43879E-4	2875	14643	3811	21206	over
GO:0051188	cofactor biosynthetic process	0.0263274	0.280437	6.32943E-4	114	429	6572	35420	over
GO:0043167	ion binding	0.0272388	0.300826	7.18938E-4	851	4068	5835	31781	over
GO:0009266	response to temperature stimulus	0.0272388	0.301157	7.20224E-4	162	651	6524	35198	over
GO:0010287	plastoglobule	0.0272388	0.308021	7.27149E-4	9	9	6677	35840	over
GO:0005975	carbohydrate metabolic process	0.0272388	0.310049	7.39667E-4	429	1943	6257	33906	over
GO:0016620	oxidoreductase activity, acting on the aldehyde or oxo group of donors, NAD or NADP as acceptor	0.0272388	0.313676	7.43047E-4	47	142	6639	35707	over
GO:0042803	protein homodimerization activity	0.0272388	0.317721	7.76816E-4	22	48	6664	35801	over
GO:0051287	NAD binding	0.0272388	0.321709	7.97482E-4	53	167	6633	35682	over
GO:0004240	mitochondrial processing peptidase activity	0.028181	0.33815	8.20806E-4	6	3	6680	35846	over
GO:0019253	reductive pentose-phosphate cycle	0.028181	0.340121	8.32235E-4	41	119	6645	35730	over
GO:0016052	carbohydrate catabolic process	0.0288924	0.356376	8.53909E-4	128	497	6558	35352	over
GO:0044275	cellular carbohydrate catabolic process	0.0288924	0.356376	8.53909E-4	128	497	6558	35352	over
GO:0005488	binding	0.031121	0.382708	9.17707E-4	3126	16017	3560	19832	over
GO:0006563	L-serine metabolic process	0.0311584	0.387854	9.24247E-4	32	85	6654	35764	over
GO:0048586	regulation of long-day photoperiodism, flowering	0.0311688	0.395403	9.48262E-4	10	12	6676	35837	over
GO:0010025	wax biosynthetic process	0.0311688	0.402098	9.90651E-4	19	39	6667	35810	over
GO:0010166	wax metabolic process	0.0311688	0.402098	9.90651E-4	19	39	6667	35810	over
GO:0015994	chlorophyll metabolic process	0.0313012	0.408054	0.00104476	37	105	6649	35744	over
GO:0004332	fructose-bisphosphate aldolase activity	0.0313624	0.41328	0.00106127	18	36	6668	35813	over
GO:0048580	regulation of post-embryonic development	0.0319302	0.423539	0.00110475	11	15	6675	35834	over
GO:0044262	cellular carbohydrate metabolic process	0.033351	0.451193	0.00120454	232	993	6454	34856	over
GO:0003746	translation elongation factor activity	0.033351	0.45414	0.00122112	44	134	6642	35715	over
GO:0043467	regulation of generation of precursor metabolites and energy	0.033351	0.46191	0.00124846	19	40	6667	35809	over
GO:0042548	regulation of photosynthesis, light reaction	0.033351	0.46191	0.00124846	19	40	6667	35809	over
GO:0010109	regulation of photosynthesis	0.033351	0.46191	0.00124846	19	40	6667	35809	over
GO:0004930	G-protein coupled receptor activity	0.033351	0.466235	0.00125176	14	24	6672	35825	over
GO:0046872	metal ion binding	0.033351	0.469395	0.00128509	817	3920	5869	31929	over
GO:0009408	response to heat	0.033888	0.479214	0.0013607	75	266	6611	35583	over
GO:0015297	antiporter activity	0.0394345	0.536558	0.00153527	45	140	6641	35709	over
GO:0006790	sulfur metabolic process	0.0414594	0.5591	0.0016726	65	225	6621	35624	over
GO:0019684	photosynthesis, light reaction	0.0426899	0.591605	0.00176739	63	217	6623	35632	over

GO:0005222	intracellular cAMP activated cation channel activity	0.0426899	0.608853	0.0017785	6	4	6680	35845	over
GO:0048582	positive regulation of post-embryonic development	0.0426899	0.608853	0.0017785	6	4	6680	35845	over
GO:0048584	positive regulation of response to stimulus	0.0426899	0.608853	0.0017785	6	4	6680	35845	over
GO:0043855	cyclic nucleotide-gated ion channel activity	0.0426899	0.608853	0.0017785	6	4	6680	35845	over
GO:0005221	intracellular cyclic nucleotide activated cation channel activity	0.0426899	0.608853	0.0017785	6	4	6680	35845	over
GO:0048578	positive regulation of long-day photoperiodism, flowering	0.0426899	0.608853	0.0017785	6	4	6680	35845	over
GO:0009853	photorespiration	0.0426899	0.609517	0.00178045	41	125	6645	35724	over
GO:0019320	hexose catabolic process	0.0426899	0.626623	0.00187699	108	418	6578	35431	over
GO:0006007	glucose catabolic process	0.0426899	0.626623	0.00187699	108	418	6578	35431	over
GO:0046365	monosaccharide catabolic process	0.0426899	0.626623	0.00187699	108	418	6578	35431	over
GO:0019867	outer membrane	0.0426899	0.626794	0.00187985	57	192	6629	35657	over
GO:0004312	fatty-acid synthase activity	0.0426899	0.627994	0.00189419	23	56	6663	35793	over
GO:0016903	oxidoreductase activity, acting on the aldehyde or oxo group of donors	0.0426899	0.629906	0.00192551	56	188	6630	35661	over
GO:0042651	thylakoid membrane	0.0426899	0.633362	0.00195159	74	266	6612	35583	over
GO:0018298	protein-chromophore linkage	0.043866	0.647251	0.00211661	22	53	6664	35796	over
GO:0006564	L-serine biosynthetic process	0.0465918	0.673202	0.0022816	13	23	6673	35826	over
GO:0032787	monocarboxylic acid metabolic process	0.0492487	0.697152	0.00244744	163	679	6523	35170	over
GO:0006006	glucose metabolic process	0.0495857	0.706263	0.00257577	113	446	6573	35403	over
GO:0006555	methionine metabolic process	0.0495857	0.7079	0.0026012	38	116	6648	35733	over
GO:0016709	oxidoreductase activity, acting on paired donors, with incorporation or reduction of molecular oxygen, NADH or NADPH as one donor, and incorporation of one atom of oxygen	0.0495857	0.710589	0.00263039	20	47	6666	35802	over

AvAG

GOSSIP
Tests for all terms in Gene Ontology whether it is enriched in a test group when compared to a reference group using Fisher's excact test with Multiple Testing.
Pub: Biological Profiling of Gene Groups utilizing Gene Ontology A Statistical Framework
Poster: GOSSIP: Biological Profiling of Gene Groups utilizing Gene Ontology
by Nils Blthgen, Karsten Brand, Hanspeter Herzel, Dieter Beule

GO Term	Name	FDR	FWER	single test p-Value	# in test group	# in reference group	# non annot test	# non annot reference group	Over/Under
GO:0048037	cofactor binding	3.57554E-7	8.93884E-8	1.92797E-10	179	796	4749	36811	over
GO:0050662	coenzyme binding	6.35933E-5	3.85052E-5	1.18475E-7	131	590	4797	37017	over
GO:0016829	lyase activity	6.35933E-5	4.76938E-5	1.35653E-7	200	1010	4728	36597	over
GO:0019752	carboxylic acid metabolic process	1.50036E-4	1.84669E-4	4.87504E-7	322	1821	4606	35786	over
GO:0006082	organic acid metabolic process	1.50036E-4	1.87528E-4	5.08983E-7	322	1822	4606	35785	over
GO:0006091	generation of precursor metabolites and energy	1.62319E-4	2.43448E-4	6.5305E-7	344	1974	4584	35633	over
GO:0008152	metabolic process	5.13556E-4	8.98319E-4	2.27092E-6	2595	18493	2333	19114	over
GO:0009056	catabolic process	0.0010633	0.00212435	5.48202E-6	277	1580	4651	36027	over
GO:0044248	cellular catabolic process	0.00134987	0.00303261	7.91032E-6	271	1549	4657	36058	over
GO:0006118	electron transport	0.00280309	0.00698324	1.70967E-5	240	1362	4688	36245	over
GO:0016491	oxidoreductase activity	0.00286657	0.00785209	1.97729E-5	528	3338	4400	34269	over
GO:0015976	carbon utilization	0.00306451	0.00987053	2.28277E-5	54	210	4874	37397	over
GO:0044237	cellular metabolic process	0.00306451	0.00991024	2.30307E-5	2158	15319	2770	22288	over
GO:0009507	chloroplast	0.00314061	0.0114025	2.45441E-5	530	3360	4398	34247	over
GO:0003824	catalytic activity	0.00314061	0.0140295	3.17956E-5	2276	16233	2652	21374	over
GO:0030170	pyridoxal phosphate binding	0.00314061	0.0141354	3.24636E-5	45	165	4883	37442	over
GO:0006096	glycolysis	0.00314061	0.0144877	3.3884E-5	76	338	4852	37269	over
GO:0009628	response to abiotic stimulus	0.00314061	0.0146981	3.48246E-5	329	1979	4599	35628	over
GO:0006950	response to stress	0.00314061	0.0148072	3.53988E-5	380	2329	4548	35278	over
GO:0010287	plastoglobule	0.00596039	0.0293624	6.78935E-5	9	9	4919	37598	over
GO:0009533	chloroplast stromal thylakoid	0.0075271	0.0402927	9.75847E-5	8	7	4920	37600	over
GO:0015979	photosynthesis	0.0075271	0.0405541	9.91313E-5	112	571	4816	37036	over
GO:0019842	vitamin binding	0.00799785	0.0449466	1.1242E-4	62	271	4866	37336	over
GO:0006519	amino acid and derivative metabolic process	0.00929365	0.0542363	1.25568E-4	244	1439	4684	36168	over
GO:0044262	cellular carbohydrate metabolic process	0.00958507	0.0603919	1.37111E-4	184	1041	4744	36566	over
GO:0009266	response to temperature stimulus	0.00958507	0.0604025	1.37127E-4	129	684	4799	36923	over
GO:0016052	carbohydrate catabolic process	0.00988625	0.066965	1.52084E-4	103	522	4825	37085	over
GO:0044275	cellular carbohydrate catabolic process	0.00988625	0.066965	1.52084E-4	103	522	4825	37085	over

GO:0016830	carbon-carbon lyase activity	0.00988625	0.0691678	1.59862E-4	85	412	4843	37195	over
GO:0009308	amine metabolic process	0.0145479	0.106982	2.42E-4	200	1161	4728	36446	over
GO:0006007	glucose catabolic process	0.0145479	0.115707	2.65371E-4	88	438	4840	37169	over
GO:0046365	monosaccharide catabolic process	0.0145479	0.115707	2.65371E-4	88	438	4840	37169	over
GO:0019320	hexose catabolic process	0.0145479	0.115707	2.65371E-4	88	438	4840	37169	over
GO:0019685	photosynthesis, dark reaction	0.0145479	0.119365	2.7166E-4	36	135	4892	37472	over
GO:0009570	chloroplast stroma	0.0145479	0.119528	2.72052E-4	54	235	4874	37372	over
GO:0050660	FAD binding	0.0149773	0.136764	3.19715E-4	42	169	4886	37438	over
GO:0009532	plastid stroma	0.0149773	0.138679	3.29128E-4	61	278	4867	37329	over
GO:0006563	L-serine metabolic process	0.0149773	0.142759	3.32985E-4	27	90	4901	37517	over
GO:0005221	intracellular cyclic nucleotide activated cation channel activity	0.0149773	0.149625	3.34019E-4	6	4	4922	37603	over
GO:0043855	cyclic nucleotide-gated ion channel activity	0.0149773	0.149625	3.34019E-4	6	4	4922	37603	over
GO:0005222	intracellular cAMP activated cation channel activity	0.0149773	0.149625	3.34019E-4	6	4	4922	37603	over
GO:0016835	carbon-oxygen lyase activity	0.0149773	0.15123	3.40422E-4	69	326	4859	37281	over
GO:0006520	amino acid metabolic process	0.0149773	0.151494	3.41004E-4	178	1023	4750	36584	over
GO:0006006	glucose metabolic process	0.0149773	0.151899	3.45451E-4	92	467	4836	37140	over
GO:0015977	carbon utilization by fixation of carbon dioxide	0.0168885	0.179237	4.01983E-4	45	188	4883	37419	over
GO:0005996	monosaccharide metabolic process	0.0168885	0.179897	4.09926E-4	119	641	4809	36966	over
GO:0051287	NAD binding	0.0168885	0.179996	4.10527E-4	43	177	4885	37430	over
GO:0046164	alcohol catabolic process	0.0169452	0.184007	4.38298E-4	88	446	4840	37161	over
GO:0016746	transferase activity, transferring acyl groups	0.0181387	0.199251	4.66168E-4	105	554	4823	37053	over
GO:0016620	oxidoreductase activity, acting on the aldehyde or oxo group of donors, NAD or NADP as acceptor	0.0186893	0.208342	4.88511E-4	38	151	4890	37456	over
GO:0051186	cofactor metabolic process	0.0194027	0.219167	5.29753E-4	153	868	4775	36739	over
GO:0016836	hydro-lyase activity	0.0200725	0.231131	5.79005E-4	48	209	4880	37398	over
GO:0016832	aldehyde-lyase activity	0.0200725	0.23748	6.09763E-4	19	55	4909	37552	over
GO:0006066	alcohol metabolic process	0.0200725	0.24339	6.37099E-4	137	767	4791	36840	over
GO:0044265	cellular macromolecule catabolic process	0.0200725	0.243718	6.38842E-4	168	972	4760	36635	over
GO:0010205	photoinhibition	0.0200725	0.248773	6.55445E-4	12	25	4916	37582	over
GO:0043155	negative regulation of photosynthesis, light reaction	0.0200725	0.248773	6.55445E-4	12	25	4916	37582	over
GO:0019253	reductive pentose-phosphate cycle	0.0213987	0.268587	6.96562E-4	33	127	4895	37480	over
GO:0008415	acyltransferase activity	0.0213987	0.270685	7.14861E-4	94	492	4834	37115	over
GO:0009922	fatty acid elongase activity	0.0297469	0.359968	8.61578E-4	5	3	4923	37604	over
GO:0005975	carbohydrate metabolic process	0.0320634	0.386766	0.00101343	323	2049	4605	35558	over
GO:0008886	glyceraldehyde-3-phosphate dehydrogenase (NADP+) activity	0.0347834	0.421722	0.0011191	8	12	4920	37595	over
GO:0006807	nitrogen compound metabolic process	0.0347834	0.421832	0.00112059	209	1264	4719	36343	over
GO:0016747	transferase activity, transferring groups other than amino-acyl groups	0.038724	0.461977	0.00123239	94	502	4834	37105	over
GO:0015297	antiporter activity	0.038724	0.46886	0.00125088	36	149	4892	37458	over
GO:0019318	hexose metabolic process	0.038724	0.472188	0.00128609	108	593	4820	37014	over
GO:0005488	binding	0.0393488	0.482745	0.00134575	2317	16826	2611	20781	over
GO:0042732	D-xylose metabolic process	0.0393488	0.491161	0.00142008	12	28	4916	37579	over
GO:0004312	fatty-acid synthase activity	0.0393488	0.492798	0.00142788	19	60	4909	37547	over
GO:0009409	response to cold	0.0401281	0.504569	0.00144987	83	435	4845	37172	over
GO:0044238	primary metabolic process	0.0464193	0.561351	0.00160354	2126	15392	2802	22215	over

DvAD

Tests for all terms in Gene Ontology whether it is enriched in a test group when compared to a reference group using Fisher's excact test with Multiple Testing.
Pub: Biological Profiling of Gene Groups utilizing Gene Ontology A Statistical Framework
Poster: GOSSIP: Biological Profiling of Gene Groups utilizing Gene Ontology
by Nils Blthgen, Karsten Brand, Hanspeter Herzel, Dieter Beule

GO Term	Name	FDR	FWER	single test p-Value	# in test group	# in reference group	# non annot test	# non annot reference group	Over/Under
GO:0006091	generation of precursor metabolites and energy	1.21646E-4	3.04111E-5	8.57711E-8	313	2005	4010	36207	over
GO:0048037	cofactor binding	1.24808E-4	6.24019E-5	1.79669E-7	150	825	4173	37387	over
GO:0016829	lyase activity	0.00470769	0.00352454	8.87903E-6	170	1040	4153	37172	over
GO:0030170	pyridoxal phosphate binding	0.00636436	0.00634416	1.48885E-5	42	168	4281	38044	over
GO:0006118	electron transport	0.00641402	0.00798548	1.93849E-5	214	1388	4109	36824	over
GO:0050660	FAD binding	0.0098461	0.0146607	3.76632E-5	41	170	4282	38042	over

GO:0016491	oxidoreductase activity	0.017554	0.0329928	7.08289E-5	463	3403	3860	34809	over
GO:0006790	sulfur metabolic process	0.017554	0.0344992	7.53786E-5	51	239	4272	37973	over
GO:0050662	coenzyme binding	0.0226411	0.0543156	1.11324E-4	105	616	4218	37596	over
GO:0016832	aldehyde-lyase activity	0.0226411	0.0550312	1.13267E-4	19	55	4304	38157	over
GO:0030130	clathrin coat of trans-Golgi network vesicle	0.0254225	0.0746985	1.65908E-4	9	13	4314	38199	over
GO:0042803	protein homodimerization activity	0.0254225	0.0749935	1.6633E-4	18	52	4305	38160	over
GO:0009507	chloroplast	0.0254225	0.0793032	1.86722E-4	461	3429	3862	34783	over
GO:0019842	vitamin binding	0.0300306	0.0997737	2.21652E-4	55	278	4268	37934	over
GO:0009570	chloroplast stroma	0.0303743	0.107658	2.5354E-4	49	240	4274	37972	over
GO:0015979	photosynthesis	0.0309881	0.12692	3.13128E-4	98	585	4225	37627	over
GO:0007030	Golgi organization and biogenesis	0.0309881	0.13363	3.22617E-4	6	5	4317	38207	over
GO:0016830	carbon-carbon lyase activity	0.0309881	0.146438	3.4683E-4	75	422	4248	37790	over
GO:0009532	plastid stroma	0.0309881	0.146904	3.49823E-4	55	284	4268	37928	over
GO:0016837	carbon-oxygen lyase activity, acting on polysaccharides	0.0309881	0.150147	3.59952E-4	16	46	4307	38166	over
GO:0030570	pectate lyase activity	0.0309881	0.150147	3.59952E-4	16	46	4307	38166	over
GO:0051186	cofactor metabolic process	0.039037	0.193226	4.72821E-4	137	884	4186	37328	over
GO:0030660	Golgi-associated vesicle membrane	0.0392202	0.224935	5.14549E-4	10	20	4313	38192	over
GO:0012510	trans-Golgi network transport vesicle membrane	0.0392202	0.224935	5.14549E-4	10	20	4313	38192	over
GO:0030658	transport vesicle membrane	0.0392202	0.224935	5.14549E-4	10	20	4313	38192	over
GO:0008152	metabolic process	0.0392202	0.225039	5.16169E-4	2246	18842	2077	19370	over
GO:0005488	binding	0.0416088	0.244876	5.73894E-4	2047	17096	2276	21116	over
GO:0009308	amine metabolic process	0.047557	0.283171	6.91404E-4	175	1186	4148	37026	over

AvD

Tests for all terms in Gene Ontology whether it is enriched in a test group when compared to a reference group using Fisher's excact test with Multiple Testing.
Pub: Biological Profiling of Gene Groups utilizing Gene Ontology A Statistical Framework
Poster: GOSSIP: Biological Profiling of Gene Groups utilizing Gene Ontology
by Nils Blthgen, Karsten Brand, Hanspeter Herzel, Dieter Beule

GO Term	Name	FDR	FWER	single test p-Value	# in test group	# in reference group	# non annot test	# non annot reference group	Over/Under
GO:0016829	lyase activity	1.29319E-6	3.23297E-7	9.90259E-10	269	941	6417	34908	over
GO:0048037	cofactor binding	2.35458E-6	1.17729E-6	3.57739E-9	222	753	6464	35096	over
GO:0006091	generation of precursor metabolites and energy	4.90566E-6	3.67924E-6	1.14387E-8	463	1855	6223	33994	over
GO:0050662	coenzyme binding	1.33985E-4	1.50016E-4	4.07165E-7	164	557	6522	35292	over
GO:0019752	carboxylic acid metabolic process	1.33985E-4	1.82127E-4	4.87881E-7	420	1723	6266	34126	over
GO:0006082	organic acid metabolic process	1.33985E-4	2.03113E-4	5.13474E-7	420	1724	6266	34125	over
GO:0009628	response to abiotic stimulus	1.33985E-4	2.34447E-4	6.09351E-7	448	1860	6238	33989	over
GO:0006118	electron transport	0.00104648	0.00209078	5.48119E-6	317	1285	6369	34564	over
GO:0003824	catalytic activity	0.00141278	0.00376743	9.82172E-6	3069	15440	3617	20409	over
GO:0030570	pectate lyase activity	0.00141278	0.0038776	1.02744E-5	24	38	6662	35811	over
GO:0016837	carbon-oxygen lyase activity, acting on polysaccharides	0.00141278	0.0038776	1.02744E-5	24	38	6662	35811	over
GO:0006950	response to stress	0.00169086	0.00527298	1.30697E-5	505	2204	6181	33645	over
GO:0015979	photosynthesis	0.00169086	0.00587612	1.45583E-5	149	534	6537	35315	over
GO:0008152	metabolic process	0.00169086	0.00590054	1.48543E-5	3472	17616	3214	18233	over
GO:0016835	carbon-oxygen lyase activity	0.002224	0.00830532	1.78495E-5	94	301	6592	35548	over
GO:0016830	carbon-carbon lyase activity	0.00241475	0.0103528	2.41606E-5	113	384	6573	35465	over
GO:0044237	cellular metabolic process	0.00241475	0.0103691	2.43046E-5	2898	14579	3788	21270	over
GO:0009507	chloroplast	0.00241475	0.0108076	2.55532E-5	701	3189	5985	32660	over
GO:0015976	carbon utilization	0.00306351	0.0144463	3.37497E-5	67	197	6619	35652	over
GO:0006096	glycolysis	0.00354918	0.0175894	4.30167E-5	96	318	6590	35531	over
GO:0009056	catabolic process	0.00436928	0.0226777	5.66265E-5	353	1504	6333	34345	over
GO:0044248	cellular catabolic process	0.00521616	0.0287752	6.63439E-5	346	1474	6340	34375	over
GO:0006520	amino acid metabolic process	0.00521616	0.0295477	7.04163E-5	238	963	6448	34886	over
GO:0009308	amine metabolic process	0.00731095	0.0443535	9.65193E-5	265	1096	6421	34753	over
GO:0016832	aldehyde-lyase activity	0.00731095	0.0446656	9.79623E-5	25	49	6661	35800	over
GO:0009570	chloroplast stroma	0.00810778	0.0513364	1.10456E-4	70	219	6616	35630	over
GO:0030170	pyridoxal phosphate binding	0.00923482	0.0604326	1.27889E-4	54	156	6632	35693	over

GO:0046483	heterocycle metabolic process	0.00928837	0.0629506	1.35847E-4	95	326	6591	35523	over
GO:0009532	plastid stroma	0.010164	0.0710401	1.63157E-4	79	260	6607	35589	over
GO:0016491	oxidoreductase activity	0.0129929	0.092851	2.22419E-4	685	3181	6001	32668	over
GO:0050896	response to stimulus	0.0145404	0.1097	2.55662E-4	999	4779	5687	31070	over
GO:0019685	photosynthesis, dark reaction	0.0145404	0.109815	2.55713E-4	45	126	6641	35723	over
GO:0050660	FAD binding	0.0148231	0.115112	2.75337E-4	53	158	6633	35691	over
GO:0009314	response to radiation	0.0155255	0.134103	3.18105E-4	189	762	6497	35087	over
GO:0006519	amino acid and derivative metabolic process	0.0155255	0.135556	3.26602E-4	316	1367	6370	34482	over
GO:0006778	porphyrin metabolic process	0.0155255	0.139605	3.42272E-4	46	132	6640	35717	over
GO:0033013	tetrapyrrole metabolic process	0.0155255	0.139605	3.42272E-4	46	132	6640	35717	over
GO:0015977	carbon utilization by fixation of carbon dioxide	0.0155255	0.139909	3.45571E-4	57	176	6629	35673	over
GO:0051186	cofactor metabolic process	0.0155255	0.141909	3.5215E-4	201	820	6485	35029	over
GO:0044435	plastid part	0.0155255	0.145499	3.59329E-4	178	713	6508	35136	over
GO:0044434	chloroplast part	0.0155255	0.147124	3.63631E-4	165	653	6521	35196	over
GO:0015995	chlorophyll biosynthetic process	0.017069	0.164087	4.04844E-4	32	80	6654	35769	over
GO:0044265	cellular macromolecule catabolic process	0.0186682	0.181834	4.47479E-4	221	919	6465	34930	over
GO:0009416	response to light stimulus	0.0193655	0.191867	4.65593E-4	186	755	6500	35094	over
GO:0006779	porphyrin biosynthetic process	0.0218358	0.229949	5.20039E-4	38	104	6648	35745	over
GO:0033014	tetrapyrrole biosynthetic process	0.0218358	0.229949	5.20039E-4	38	104	6648	35745	over
GO:0042440	pigment metabolic process	0.0218358	0.230178	5.21194E-4	66	217	6620	35632	over
GO:0006807	nitrogen compound metabolic process	0.0218358	0.23104	5.25923E-4	278	1195	6408	34654	over
GO:0044238	primary metabolic process	0.0218358	0.234711	5.43879E-4	2875	14643	3811	21206	over
GO:0051188	cofactor biosynthetic process	0.0263274	0.280437	6.32943E-4	114	429	6572	35420	over
GO:0043167	ion binding	0.0272388	0.300826	7.18938E-4	851	4068	5835	31781	over
GO:0009266	response to temperature stimulus	0.0272388	0.301157	7.20224E-4	162	651	6524	35198	over
GO:0010287	plastoglobule	0.0272388	0.308021	7.27149E-4	9	9	6677	35840	over
GO:0005975	carbohydrate metabolic process	0.0272388	0.310049	7.39667E-4	429	1943	6257	33906	over
GO:0016620	oxidoreductase activity, acting on the aldehyde or oxo group of donors, NAD or NADP as acceptor	0.0272388	0.313676	7.43047E-4	47	142	6639	35707	over
GO:0042803	protein homodimerization activity	0.0272388	0.317721	7.76816E-4	22	48	6664	35801	over
GO:0051287	NAD binding	0.0272388	0.321709	7.97482E-4	53	167	6633	35682	over
GO:0004240	mitochondrial processing peptidase activity	0.028181	0.33815	8.20806E-4	6	3	6680	35846	over
GO:0019253	reductive pentose-phosphate cycle	0.028181	0.340121	8.32235E-4	41	119	6645	35730	over
GO:0016052	carbohydrate catabolic process	0.0288924	0.356376	8.53909E-4	128	497	6558	35352	over
GO:0044275	cellular carbohydrate catabolic process	0.0288924	0.356376	8.53909E-4	128	497	6558	35352	over
GO:0005488	binding	0.031121	0.382708	9.17707E-4	3126	16017	3560	19832	over
GO:0006563	L-serine metabolic process	0.0311584	0.387854	9.24247E-4	32	85	6654	35764	over
GO:0048586	regulation of long-day photoperiodism, flowering	0.0311688	0.395403	9.48262E-4	10	12	6676	35837	over
GO:0010025	wax biosynthetic process	0.0311688	0.402098	9.90651E-4	19	39	6667	35810	over
GO:0010166	wax metabolic process	0.0311688	0.402098	9.90651E-4	19	39	6667	35810	over
GO:0015994	chlorophyll metabolic process	0.0313012	0.408054	0.00104476	37	105	6649	35744	over
GO:0004332	fructose-bisphosphate aldolase activity	0.0313624	0.41328	0.00106127	18	36	6668	35813	over
GO:0048580	regulation of post-embryonic development	0.0319302	0.423539	0.00110475	11	15	6675	35834	over
GO:0044262	cellular carbohydrate metabolic process	0.033351	0.451193	0.00120454	232	993	6454	34856	over
GO:0003746	translation elongation factor activity	0.033351	0.45414	0.00122112	44	134	6642	35715	over
GO:0043467	regulation of generation of precursor metabolites and energy	0.033351	0.46191	0.00124846	19	40	6667	35809	over
GO:0042548	regulation of photosynthesis, light reaction	0.033351	0.46191	0.00124846	19	40	6667	35809	over
GO:0010109	regulation of photosynthesis	0.033351	0.46191	0.00124846	19	40	6667	35809	over
GO:0004930	G-protein coupled receptor activity	0.033351	0.466235	0.00125176	14	24	6672	35825	over
GO:0046872	metal ion binding	0.033351	0.469395	0.00128509	817	3920	5869	31929	over
GO:0009408	response to heat	0.033888	0.479214	0.0013607	75	266	6611	35583	over
GO:0015297	antiporter activity	0.0394345	0.536558	0.00153527	45	140	6641	35709	over
GO:0006790	sulfur metabolic process	0.0414594	0.5591	0.0016726	65	225	6621	35624	over
GO:0019684	photosynthesis, light reaction	0.0426899	0.591605	0.00176739	63	217	6623	35632	over
GO:0005222	intracellular cAMP activated cation channel activity	0.0426899	0.608853	0.0017785	6	4	6680	35845	over

GO:0048582	positive regulation of post-embryonic development	0.0426899	0.608853	0.0017785	6	4	6680	35845	over
GO:0048584	positive regulation of response to stimulus	0.0426899	0.608853	0.0017785	6	4	6680	35845	over
GO:0043855	cyclic nucleotide-gated ion channel activity	0.0426899	0.608853	0.0017785	6	4	6680	35845	over
GO:0005221	intracellular cyclic nucleotide activated cation channel activity	0.0426899	0.608853	0.0017785	6	4	6680	35845	over
GO:0048578	positive regulation of long-day photoperiodism, flowering	0.0426899	0.608853	0.0017785	6	4	6680	35845	over
GO:0009853	photorespiration	0.0426899	0.609517	0.00178045	41	125	6645	35724	over
GO:0019320	hexose catabolic process	0.0426899	0.626623	0.00187699	108	418	6578	35431	over
GO:0006007	glucose catabolic process	0.0426899	0.626623	0.00187699	108	418	6578	35431	over
GO:0046365	monosaccharide catabolic process	0.0426899	0.626623	0.00187699	108	418	6578	35431	over
GO:0019867	outer membrane	0.0426899	0.626794	0.00187985	57	192	6629	35657	over
GO:0004312	fatty-acid synthase activity	0.0426899	0.627994	0.00189419	23	56	6663	35793	over
GO:0016903	oxidoreductase activity, acting on the aldehyde or oxo group of donors	0.0426899	0.629906	0.00192551	56	188	6630	35661	over
GO:0042651	thylakoid membrane	0.0426899	0.633362	0.00195159	74	266	6612	35583	over
GO:0018298	protein-chromophore linkage	0.043866	0.647251	0.00211661	22	53	6664	35796	over
GO:0006564	L-serine biosynthetic process	0.0465918	0.673202	0.0022816	13	23	6673	35826	over
GO:0032787	monocarboxylic acid metabolic process	0.0492487	0.697152	0.00244744	163	679	6523	35170	over
GO:0006006	glucose metabolic process	0.0495857	0.706263	0.00257577	113	446	6573	35403	over
GO:0006555	methionine metabolic process	0.0495857	0.7079	0.0026012	38	116	6648	35733	over
GO:0016709	oxidoreductase activity, acting on paired donors, with incorporation or reduction of molecular oxygen, NADH or NADPH as one donor, and incorporation of one atom of oxygen	0.0495857	0.710589	0.00263039	20	47	6666	35802	over
